# Supplementary material for: GDF15 is a dynamic biomarker of the integrated stress response in the central nervous system
Source: CNS Neurosci Ther. 2024 Feb 15;30(2):e14600. doi: 10.1111/cns.14600 (PMC10867791; doi:10.1111/cns.14600)
Supplement: Supplementary file 1 — Figure S1. [file CNS-30-e14600-s007.pdf]

Figure S1:

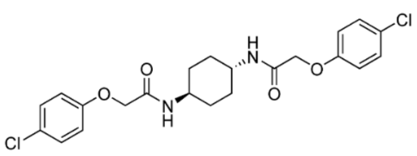

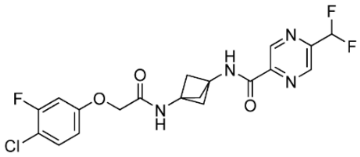

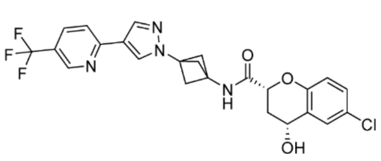

ISRIB (12 nM) 2BAct (29 nM) 2BAct-1 (2 nM)

Figure S1: Chemical structures and IC50s of ISRIB, 2BAct and 2BAct-1. Compound potency was determined in an ATF4-luciferase cell-based reporter assay..
