# Supplementary figures and images for: GDF15 is a dynamic biomarker of the integrated stress response in the central nervous system
Source: CNS Neurosci Ther. 2024 Feb 15;30(2):e14600. doi: 10.1111/cns.14600 (PMC10867791; doi:10.1111/cns.14600)

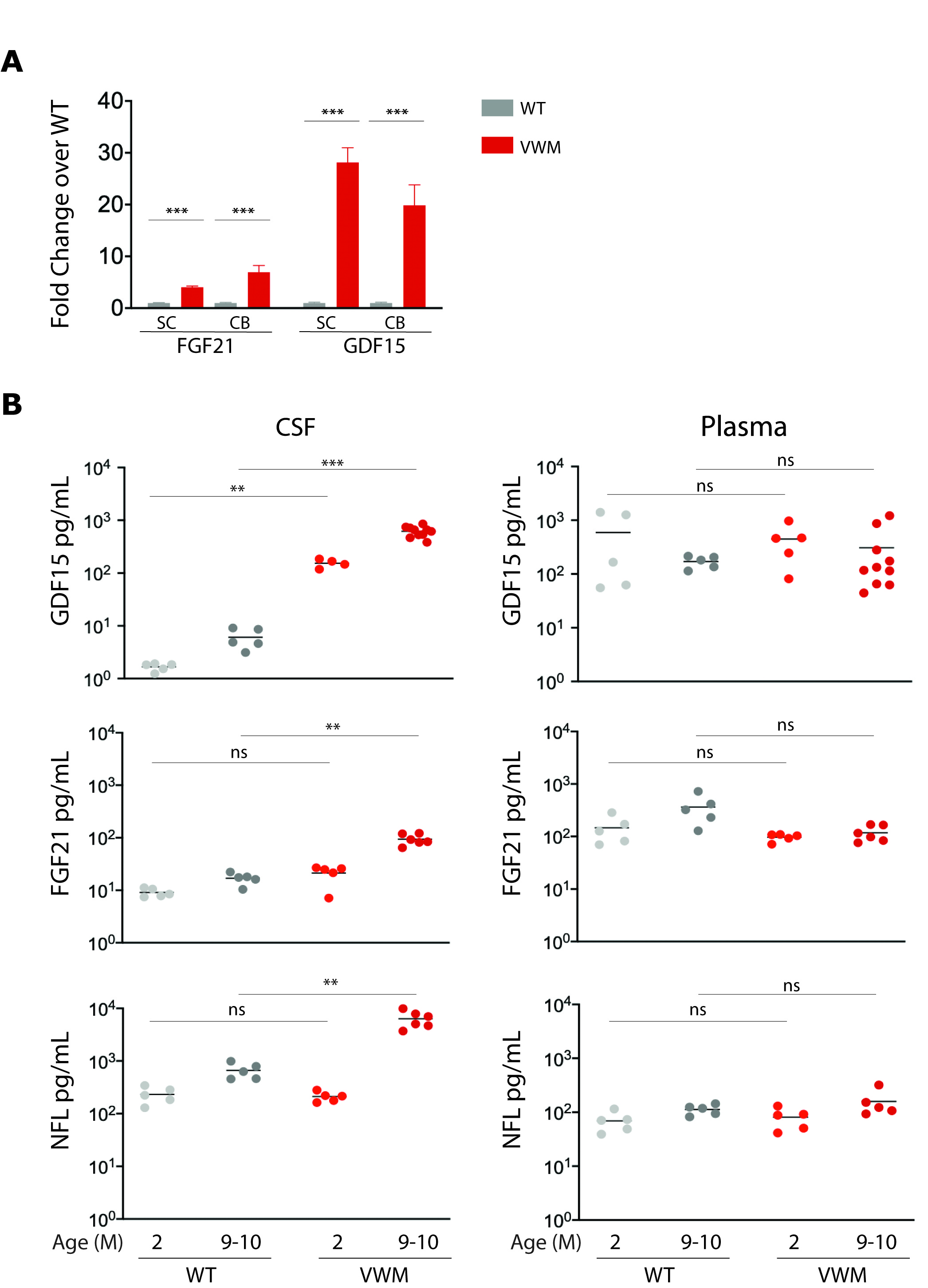

Supplement: Supplementary file 2 — Figure S2. [file CNS-30-e14600-s005.jpg]

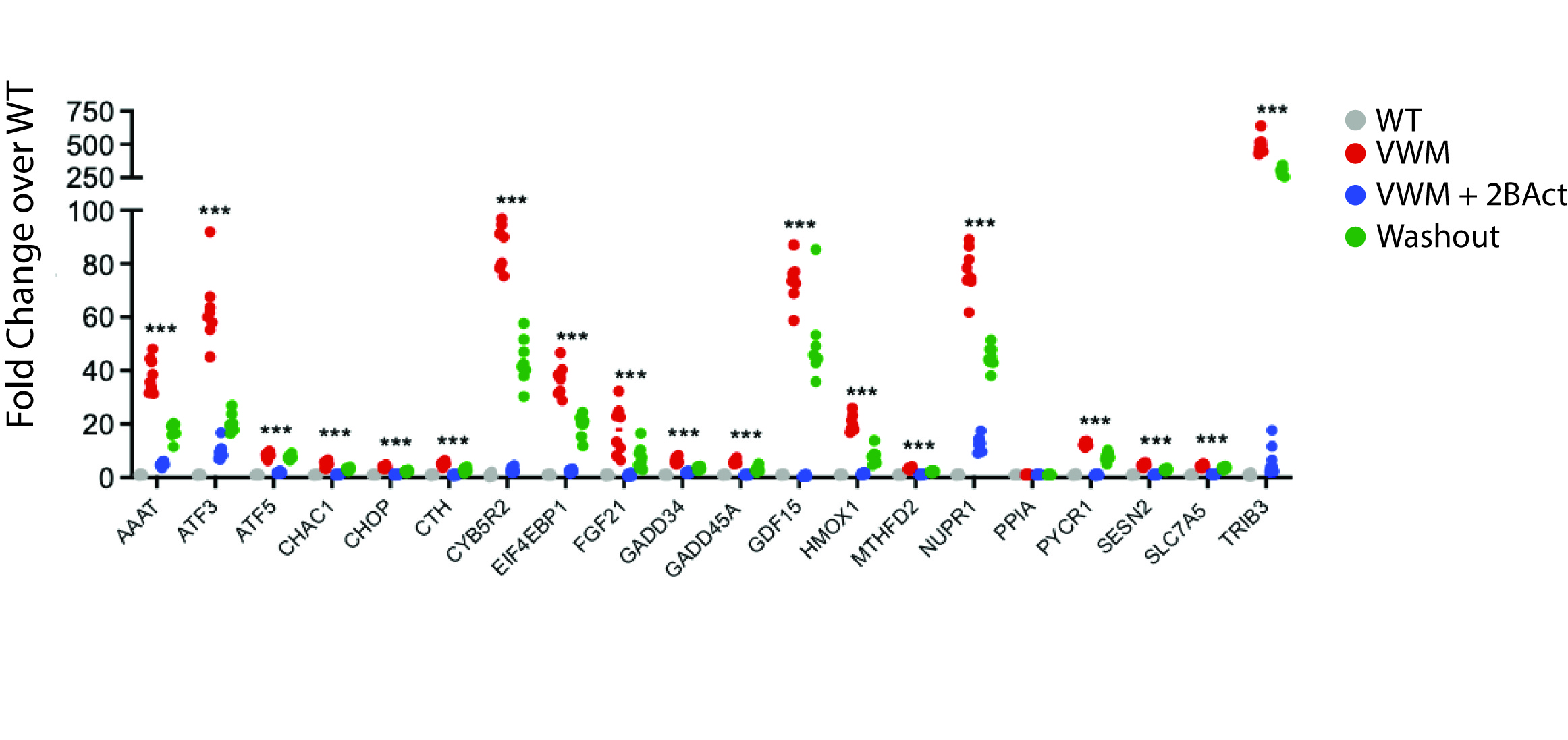

Supplement: Supplementary file 3 — Figure S3. [file CNS-30-e14600-s002.jpg]

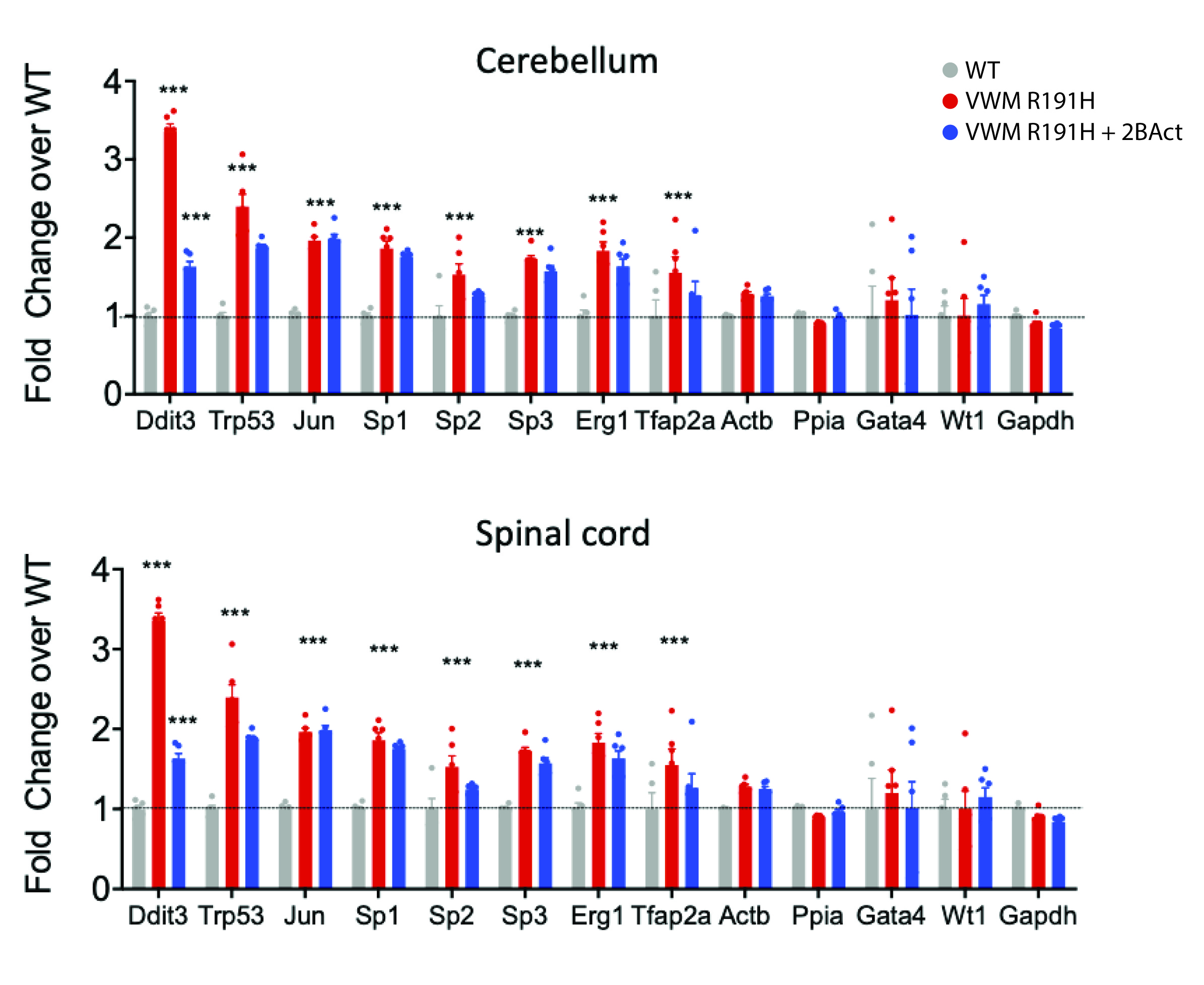

Supplement: Supplementary file 4 — Figure S4. [file CNS-30-e14600-s003.jpg]

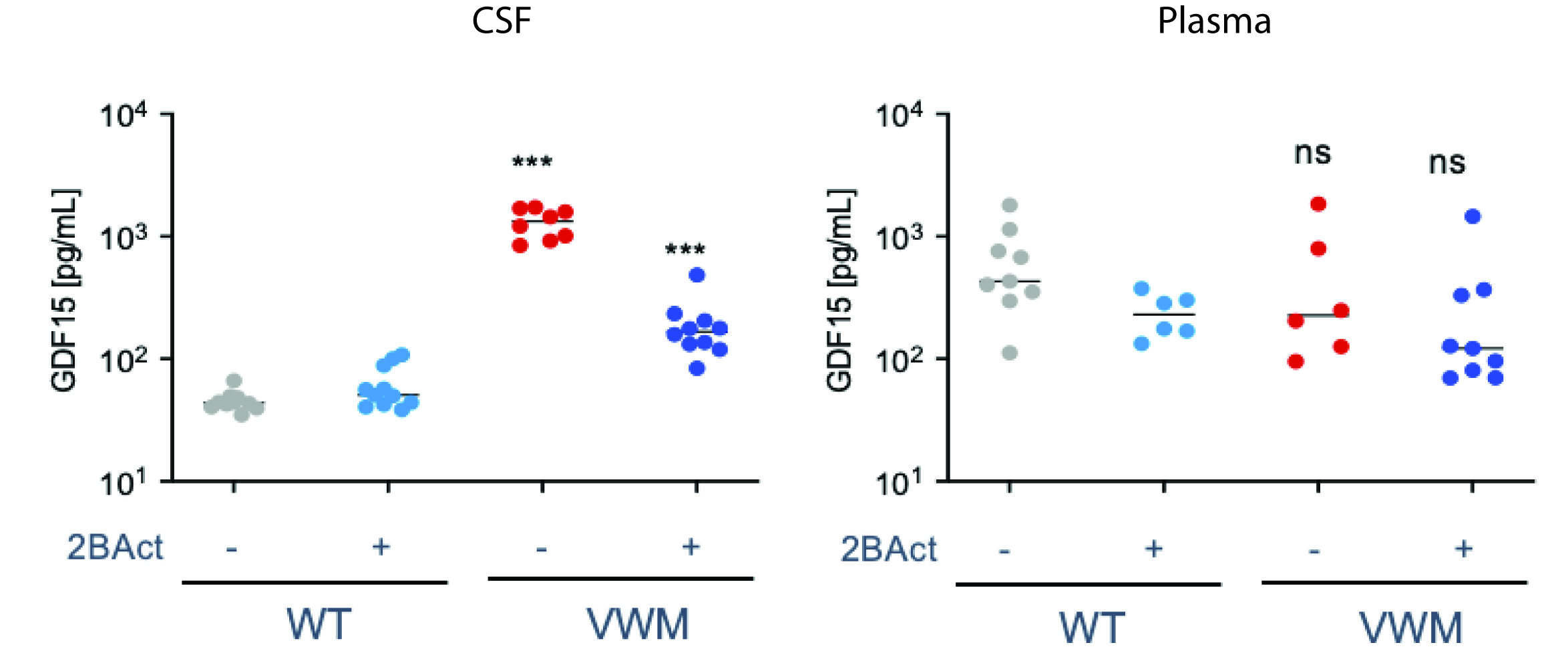

Supplement: Supplementary file 5 — Figure S5. [file CNS-30-e14600-s004.jpg]

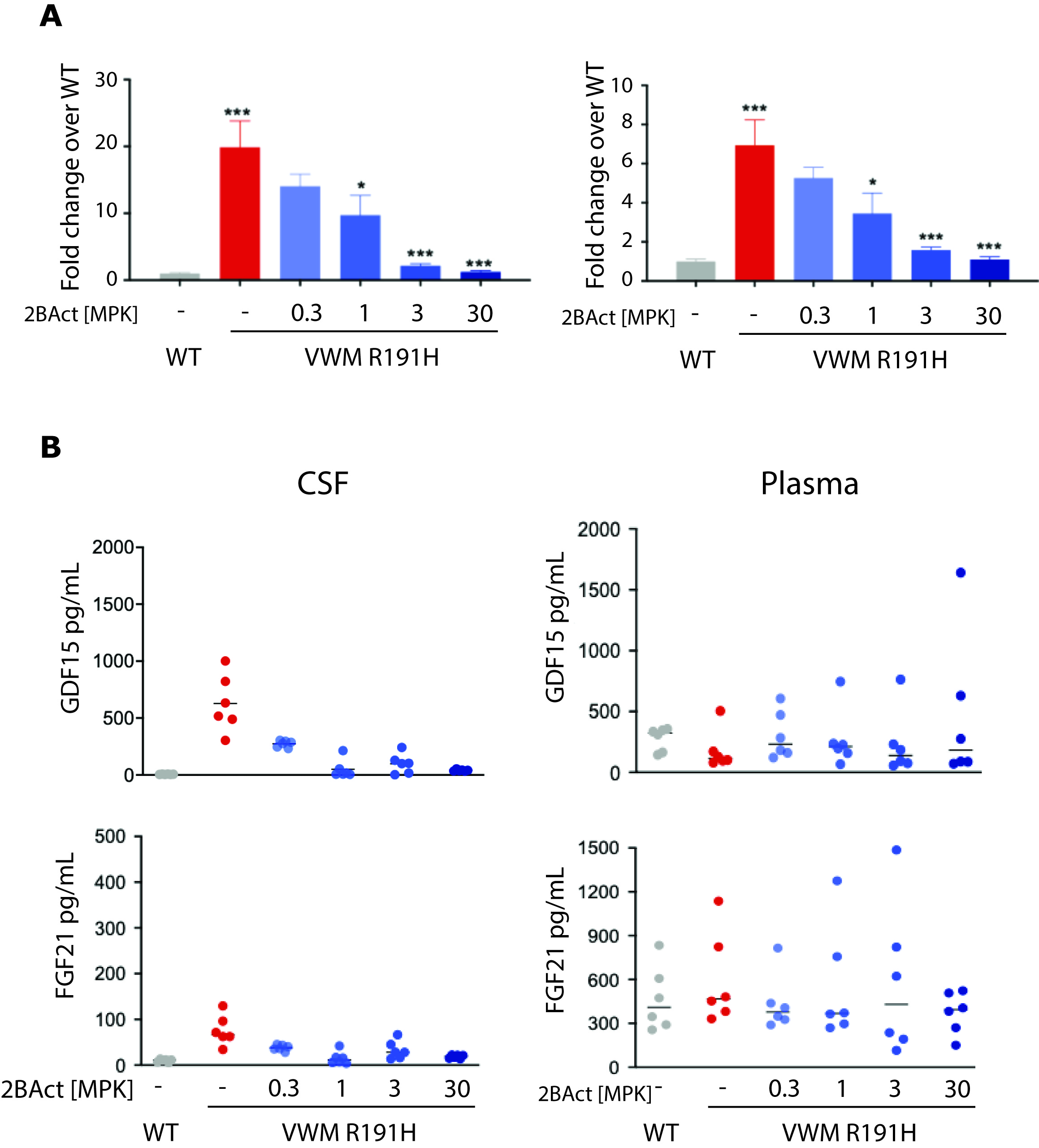

Supplement: Supplementary file 6 — Figure S6. [file CNS-30-e14600-s008.jpg]

**Table S1.** Immunoassays

**
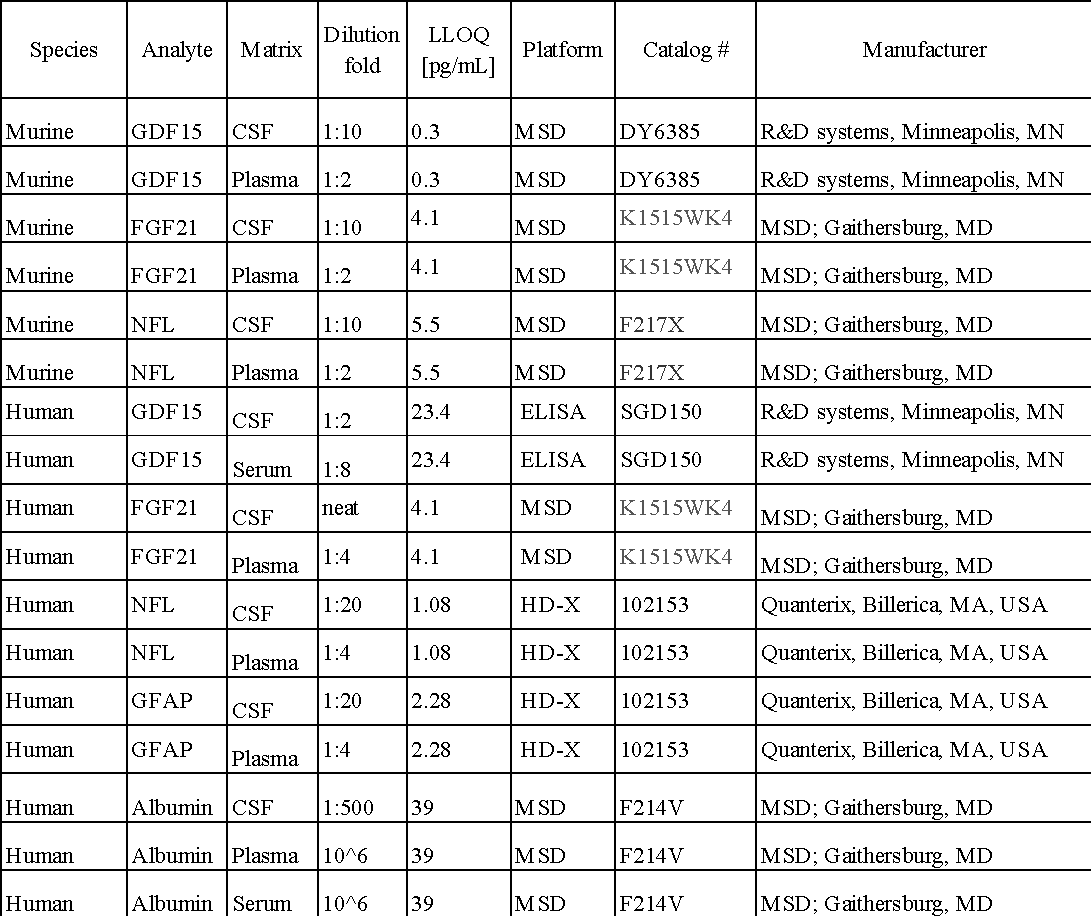
**

Supplement: Supplementary file 7 — Table S1. [file CNS-30-e14600-s009.docx]

**Table S2. Demographics and genetic information of samples from patients with VWM disease.**

**
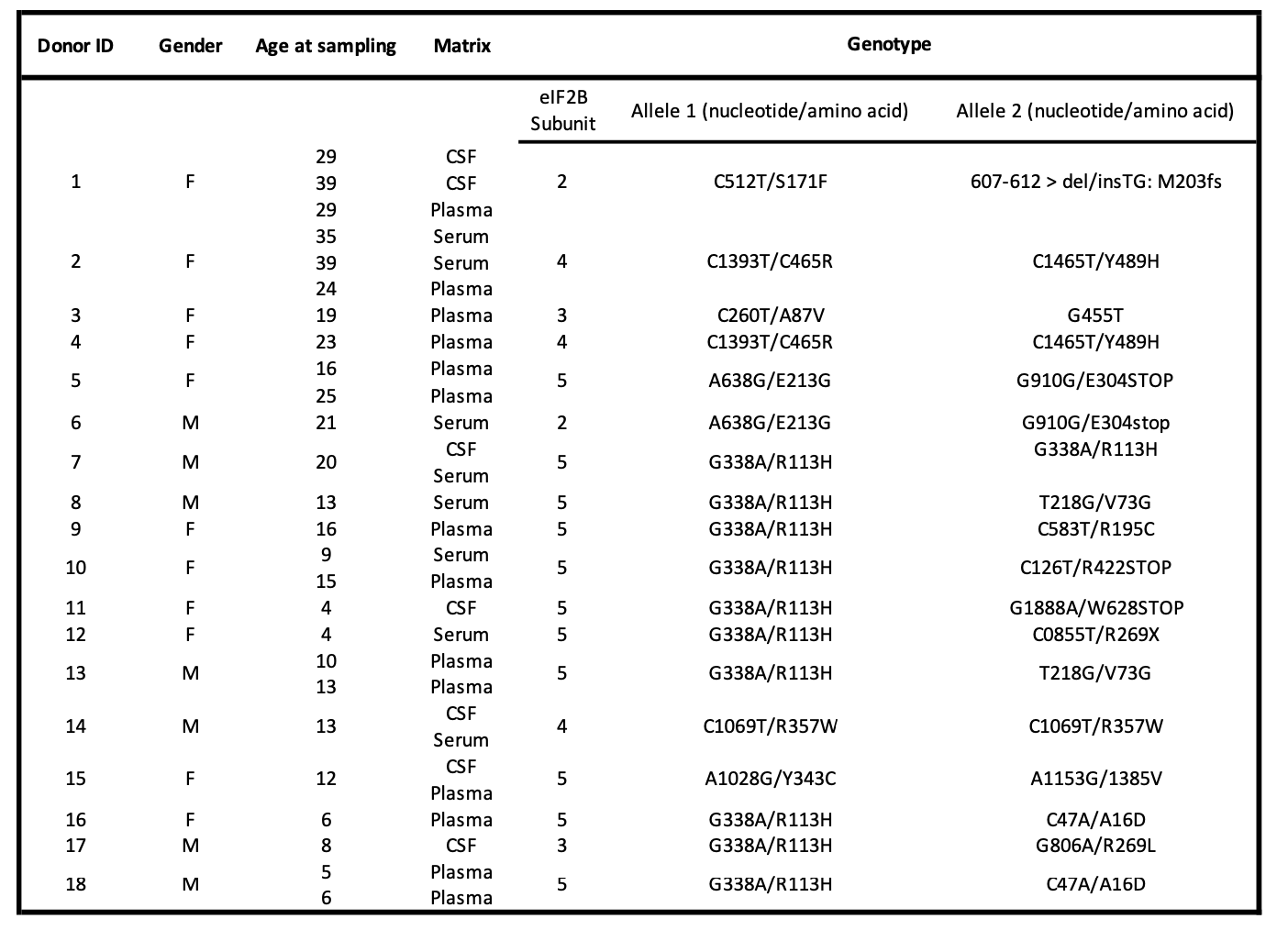
**

Supplement: Supplementary file 8 — Table S2. [file CNS-30-e14600-s001.docx]
